# A Pilot Study of Urinary Cubilin Shedding in Type 1 Diabetes Mellitus: A Potential Biomarker to Predict Nephropathy

**DOI:** 10.1155/jdr/2291735

**Published:** 2026-08-21

**Authors:** E. Ciccia, B. Zhang, T. Costacou, E. Erkan

**Affiliations:** ^1^ St. Louis Children′s Hospital, Washington University School of Medicine, St. Louis, Missouri, USA, wustl.edu; ^2^ Cincinnati Children′s Hospital, University of Cincinnati, Cincinnati, Ohio, USA, uc.edu; ^3^ Department of Epidemiology, University of Pittsburgh, Pittsburgh, Pennsylvania, USA, pitt.edu

## Abstract

Considering the low sensitivity of microalbuminuria and the increasing prevalence of diabetic nephropathy, biomarker discovery is a priority in diabetes research. We have previously reported increased urinary cubilin shedding preceding microalbuminuria in patients with Type 1 diabetes. In this study, we investigated the predictive value of urinary cubilin as a biomarker in diabetic nephropathy utilizing urine samples collected in one of the largest prospective cohort studies of Type 1 diabetes, Pittsburgh Epidemiology of Diabetes Complications. We measured urinary cubilin by ELISA in urine samples of the participants with normal kidney function and normoalbuminuria obtained at recruitment. Patients were stratified into three groups based on their disease course during a follow‐up of > 20 years. Group 1 preserved normal kidney function and normoalbuminuria, Group 2 preserved kidney function but developed macroalbuminuria, and Group 3 progressed to have a > 40% decrease in kidney function during follow‐up. We demonstrated that urinary cubilin normalized for creatinine predicted progressive diabetic nephropathy with an area under the receiver operating characteristic curve of 0.81. The urinary cubilin:creatinine ratio was marginally significant across the three groups evaluated by the Kruskal–Wallis test (*p* = 0.066). The adjusted *p* value for multiple comparisons of the urinary cubilin:creatinine ratio in comparing the group with > 40% decrease in kidney function and the other two groups was 0.05. The urine cubilin:creatinine ratio between the group with > 40% decrease in GFR and preserved kidney function was significant, with a *p* value of 0.017. We propose that urinary cubilin can be a promising biomarker to predict progressive diabetic nephropathy in patients with Type 1 diabetes. Studies involving larger patient populations are warranted to further validate urinary cubilin shedding as a novel biomarker in predicting progressive diabetic nephropathy.

## 1. Introduction

Diabetic nephropathy (DN) is the most common cause of end‐stage kidney disease (ESKD) in adults, accounting for 40% of new cases. The estimated Type 1 diabetes (T1D) prevalence per 1000 youths for those 19 years or younger increased significantly from 1.48 in 2001 to 1.93 in 2009, and to 2.15 in 2017, representing an absolute increase of 0.67 per 1000 youths and a 45.1% relative increase over 16 years in the United States [1]. Microalbuminuria (MA) is a clinical tool to monitor kidney damage and predict future cardiovascular and neurocognitive complications. Previously reported low sensitivity and advanced renal damage at the time of MA have prompted research efforts seeking alternative biomarkers to capture early molecular alterations to predict development of DN [2–4]. Although DN is primarily a glomerular disease, early markers of tubular dysfunction have been suggested as having clinical utility in the early stages of DN [5–7].

Proximal tubule epithelial cells (PTECs) possess a delicate mechanism to retrieve albumin from the glomerular filtrate via the megalin–cubilin receptor complex and amnionless [1, 2]. We reported that impaired megalin expression, resulting in decreased internalization and altered intracellular trafficking, caused urinary shedding of cubilin in both the mouse model and patients with T1D [8].

Neutrophil gelatinase–associated lipocalin (NGAL) is an indicator of kidney inflammation and PTEC injury and is widely used to predict acute kidney injury. Several studies have proposed NGAL as a valuable biomarker for the early detection of DN in T1D [9].

Elevated levels of circulating and urinary proinflammatory cytokines have been reported in T1D and Type 2 diabetes (T2D) mellitus [10]. Increased concentrations of circulating and urinary interleukin‐8 (IL‐8) and monocyte chemoattractant protein‐1(MCP‐1) correlate with urinary albumin excretion and DN [11–16].

In this pilot study, we investigated the validity and predictive value of tubular and inflammatory biomarkers IL‐8, MCP‐1, cubilin, NGAL, and vitamin D binding protein (VDBP) in a cohort of patients with T1D. We utilized urine samples of individuals diagnosed with T1D between 1950 and 1980 who were recruited into the Pittsburgh Epidemiology of Diabetes Complications (EDC), one of the largest prospective cohort studies in T1D (*n* = 658) [17]. Urine samples utilized in our study were collected at study baseline prior to the development of MA or renal insufficiency. The average follow‐up of the patients was > 20 years.

## 2. Methods

Urine samples utilized in our study were collected at the time of enrollment. All urine samples were obtained from patients with normal kidney function and urinalysis without MA.

### 2.1. Study Protocol

Study participants underwent biennial surveys and clinical examinations for 10 years, after which biennial surveys continued until 32 years of follow‐up, whereas additional examinations were conducted at Years 18, 25, and 30. Three timed urine samples (24 h, overnight, and 4 h) were collected, and urine albumin was measured by immunonephelometry. MA was defined as an albumin excretion rate (AER) of 20–200 *μ*g/min, and macroalbuminuria as an AER > 200 *μ*g/min, in at least two out of three timed urine samples. In this study, urine samples were used from participants with normal kidney function, with an estimated glomerular filtration rate (eGFR) > 90 mL/min/1.73 m^2^ and normoalbuminuria (NA) at baseline. eGFR was estimated by the Chronic Kidney Disease Epidemiology Collaboration (CKD‐EPI) creatinine formula.

Spot urine samples obtained at the time of clinic visit were used for analyses in this study. Samples were stored at −20°C, thawed at room temperature, and centrifuged at 10,000 × g for 10 min. Supernatant was removed and used for analysis. Samples were processed after the first thaw. Multiple freeze‐thaw cycles were avoided.

### 2.2. Study Population

EDC is a prospective cohort study of childhood‐onset (< 17 years) T1D. The study was initiated between 1986 and 1988 and recruited patients who had been seen within 1 year of diagnosis at Children′s Hospital of Pittsburgh (*n* = 658; mean age, 28 years; mean T1D duration, 19 years) [17].

Patients were grouped according to kidney function because a decline in eGFR is a key clinical outcome and is strongly associated with progression to ESKD.

Study groups were determined as follows:

• Group 1 (*n* = 9): Preserved kidney function during follow‐up (eGFR > 90 mL/min/1.73 m^2^).
• Group 2 (*n* = 9): In this group, patients developed macroalbuminuria characterized by urine albumin excretion > 200 *μ*g/min, but they did not exhibit significant decline in eGFR during follow‐up (eGFR > 70 mL/min/1.73 m^2^).
• Group 3 (*n* = 10): Developed progressive DN defined as > 40% decline in eGFR from baseline during follow‐up.

The sample size was small because baseline urine samples were available in the EDC for only a few patients with normal kidney function and NA.

### 2.3. Experimental Methods

All urine biomarkers were measured by ELISA (MyBioSource Cubilin assay and R&D Systems) and normalized for creatinine. Urine creatinine was measured by colorimetric ELISA (R&D). This study assessed urinary IL‐8, cubilin, MCP‐1, NGAL, and VDBP levels by ELISA across three groups of patients with childhood‐onset T1D.

### 2.4. Statistical Studies

Descriptive statistics were used to summarize the baseline characteristics of the patients. A Kruskal–Wallis test was used to compare the continuous variables among groups. Univariate and multivariate logistic regression models were used to assess associations between each urinary protein as a biomarker and decreased GFR. Mann–Whitney test was used for individual comparisons between groups. Receiver operating characteristic (ROC) analysis was conducted to assess the predictive ability of each biomarker. The area under the curve (AUC) and its 95% confidence interval (CI), sensitivity, and specificity were used to evaluate the overall performance. The optimal threshold for the biomarker which maximized Youden′s index was also reported. All analyses were performed using SAS Version 9.4 (SAS Institute Inc., Cary, North Carolina, United States). A *p* value less than 0.05 was considered statistically significant.

ROC analysis was performed to distinguish decreased GFR from normal AER + GFR. The AUC and its 95% CI were calculated using DeLong′s method.

This study was conducted in accordance with the Declaration of Helsinki, and the protocol was approved by the Institutional Review Board of the University of Pittsburgh. IRB approval number is CR19040065‐012. Written informed consent was obtained from all subjects involved in the study. We used already obtained and stored patient urine samples that were collected within the EDC cohort (consent forms from patients were collected at the time of enrollment); therefore, a separate consent was not obtained for utilization of the urine samples.

The signed consent form explicitly mentioned that the collected samples would be used for research and publication purposes.

## 3. Results and Discussion

The baseline characteristics and duration of follow‐up among three groups were comparable. Follow‐up AER was significantly elevated in the groups that developed macroalbuminuria and progressive DN (Table 1). Despite the relatively higher urine protein concentration in the progressive DN group, this value did not reach statistical significance, and there was no difference in timing of MA among groups.

**Table 1:** Urinary IL‐8, cubilin, MCP‐1, NGAL, and VDBP levels measured by ELISA.

	Preserved kidney function without albuminuria (*n* = 9)	Macroalbuminuria (albumin excretion > 200 *μ*g/min) (*n* = 9)	Progressive diabetic nephropathy (> 40% decline in GFR) (*n* = 10)
Age, median (IQR)	21.93 (16.49, 26.13)	19.74 (17.84, 25.26)	22.12 (20.97, 25.7)
Gender (M/F)	2/7	7/2	2/8
Race (W/B)	9/0	9/1	10/0
Duration	13.43 (10.56, 16.52)	9.8 (8.65, 15.36)	13.29 (9.67, 15.84)
Follow‐up (years), median (IQR)	25.2 (24.65, 25.30)	25.72 (18.15, 26.41)	24.88 (19.87, 25.32)
AER (baseline), median (IQR)	5.53 (4.54, 10.23)	9.03 (7.39, 10.20)	7.80 (5.68, 10.89)
AER (follow‐up), median (IQR)	4.84 (4.20, 12.75)	213.2 (42.30, 1139.07)	795.79 (146, 2505.28)
eGFR (baseline), median (IQR)	129.44 (109.34, 135.60)	120.20 (112.10, 134.64)	125.19 (105.07, 133.86)
eGFR (follow‐up), median (IQR)	96.31 (86.65, 104.47)	81.64 (72.75, 86.17)	27.83 (21.52, 50.84)
ACE inhibitor	9/0	9/8	10/7
Kidney transplantation	9/0	9/0	10/4
IL‐8/cre (pg/mg), median (IQR)	193.41 (92.13, 249.91)	71.07 (35.45, 273.54)	113.14 (47.45, 469.26)
NGAL/cre (pg/mg), median (IQR)	264.85 (170.53, 524.44)	80.18 (0.00, 345.00)	369.64 (51.35, 578.94)
VDBP/cre (ng/mg), median (IQR)	81.18 (2.26, 193.37)	9.55 (2.77, 70.47)	55.92 (15.62, 212.21)
MCP/cre (pg/mg), median (IQR)	1366.10 (760.10, 1873.62)	536.41 (237.60, 1939.61)	842.30 (367.79, 1082.41)
Cubilin/cre (ng/mg), median (IQR)	21.42 (16.57, 26.69)	29.41 (14.32, 42.42)	38.42 (30.29, 56.57)

*Note:* A Mann–Whitney *U* test was used for individual comparisons between the group that maintained normal eGFR (*n* = 9), the group that developed macroalbuminuria with normal eGFR (*n* = 9), and the group with a > 40% decrease in eGFR (*n* = 10) during follow‐up. Baseline demographics of the three groups were comparable. All three groups had normal eGFR and urinary albumin excretion at baseline. Follow‐up AER was higher in the groups with macroalbuminuria and normal eGFR, as well as those with decreased eGFR, in comparison to the group with preserved kidney function without albuminuria. The median values, along with the lower and upper quantiles of urinary biomarker values corrected for creatinine, are presented in this table. Multiple comparisons were performed using the Kruskal–Wallis test. A marginal difference among the three classes was observed for the urine cubilin:creatinine ratio. *p* = 0.06.

**Figure 1:**
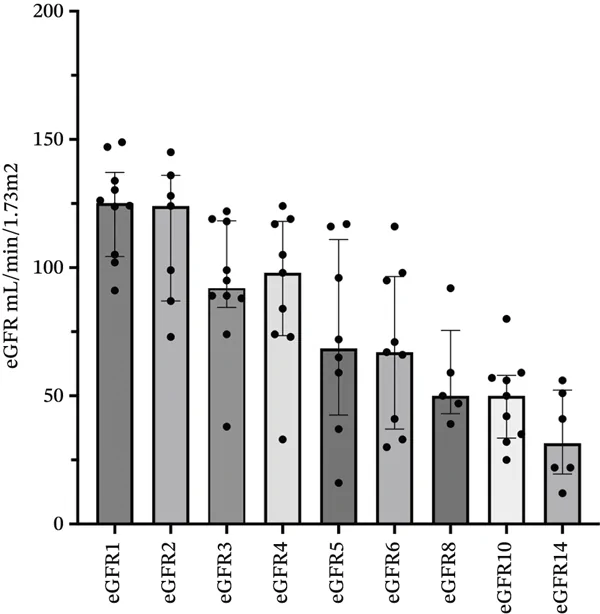
Estimated GFR decline by visit number among patients who experienced a > 40% decrease in eGFR during follow‐up.

Levels of urinary IL‐8, MCP‐1, NGAL, and VDBP did not differ among the three groups (Table 1). Raw data depicting urine ELISA measurements of potential biomarkers among groups were included in the supporting information. Urinary cubilin excretion differed marginally among the three groups evaluated by the Kruskal–Wallis test (*p* = 0.066). The adjusted *p* value for multiple comparisons of urinary cubilin in comparing the group with > 40% decline in eGFR and other groups was 0.05 (Table 1). The kidney function of participants with > 40% decline in eGFR over a 20‐year follow‐up is depicted in Figure 1.

**Figure 2:**
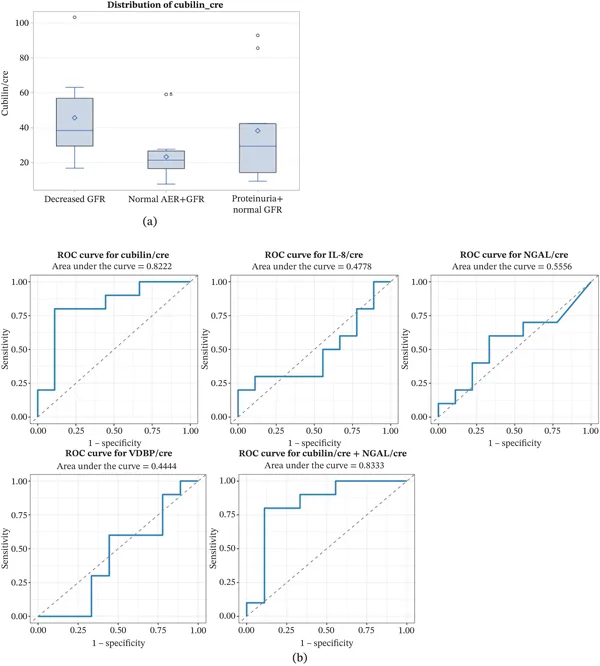
(a) Box plot showing the median and interquartile range of the urinary cubilin:creatinine ratio across the study groups. Baseline urinary cubilin:creatinine ratio was higher in the group that developed decreased eGFR compared with the group that maintained normal AER and eGFR during follow‐up (*p* = 0.017, Mann–Whitney *U* test). (b) Receiver operating characteristic (ROC) analysis was conducted to assess the predictive ability of each biomarker. The area under the curve (AUC) and its 95% confidence interval (CI), sensitivity, and specificity were used to evaluate the overall performance. The urinary cubilin:creatinine ratio demonstrated an AUC of 0.82 for predicting progressive DN. Combining the tubular injury marker NGAL with cubilin resulted in only a minimal increase in the AUC, from 0.82 to 0.83.

The*p*value for direct comparison between the group with a > 40% decrease in GFR and the group with preserved kidney function and NA at follow‐up, using the Mann–Whitney*U*test, was 0.017 (Figure 2a). Interestingly, only urinary cubilin and NGAL levels showed an increase in the group that maintained normal AER/eGFR compared with those with a decline in eGFR.

The AUC for cubilin to predict progressive DN was 0.82 (95*%*CI = 0.61, 1). The univariate and multivariate models show that the unadjusted and adjusted odds ratios are 1.07 (95*%*CI = 1, 1.06) and 1.08 (95*%*CI = 1, 1.17). The addition of NGAL did not increase AUC significantly, increasing the AUC to 0.83 (95*%*CI = 0.62, 1). A urinary cubilin value, with a cutoff value of 29.5, maximized the sensitivity and specificity (Figure 2b).

## 4. Conclusions

Cubilin is a 460 kilodalton glycosylated protein with 27 C‐terminal CUB repeats, coexpressed with megalin to facilitate albumin endocytosis in PTEC and podocytes. It does not possess a transmembrane domain and is therefore amenable to urinary shedding. We previously reported downregulation of megalin and cubilin expression in PTEC in an animal model of T1DM and urinary shedding of cubilin preceding MA in patients with T1D [8]. Megalin and cubilin shedding at the time of MA was reported in Type 1 DM; however, the cross‐sectional design of the previous studies makes it impossible to establish a causal relationship between MA and urinary receptor shedding [18]. We hypothesize that in early DN, decreased membrane expression of megalin leads to mistrafficking of cubilin, resulting in its urinary shedding due to the absence of its anchoring partner megalin.

Recently, a genetic link between variants of the cubilin gene and MA and progression in DN was demonstrated. A missense variant (I2984V) in the cubilin gene, CUBN, discovered by a genome‐wide association study (GWAS), was reported to be associated with a 41% increased risk for development of persistent MA during 20 years of follow‐up among 1304 participants with T1D [19]. A GWAS of patients with T2D demonstrated that certain CUBN allele carriers are at an increased risk of heart disease and stroke [20]. Furthermore, a genetic variant of CUBN was identified as a novel risk variant for kidney function loss in ESKD and graft loss after kidney transplantation [21]. Biallelic pathogenic CUBN gene variants were reported to cause low‐grade benign proteinuria, not requiring treatment or a kidney biopsy [22]. On the contrary, CUBN mutations can be associated with focal segmental glomerulosclerosis and glomerular basement membrane changes in isolated cases [23, 24].

In line with these data, our study showed that patients with increased urinary cubilin shedding at baseline exhibited a significant decline in kidney function. The level of proteinuria in the group with progressive kidney dysfunction was higher compared with the group with macroalbuminuria and preserved kidney function at follow‐up; however, the difference was not statistically significant which shows that the effect of urinary cubilin shedding in progression was independent of proteinuria. However, the small sample size limits the power and generalizability of our findings.

Cubilin mutation causes *Imerslund–Gräsbeck syndrome* characterized by mild tubular proteinuria and B12 malabsorption. This condition does not lead to progressive kidney dysfunction. In contrast, the strong evidence showing a clear connection between disease progression and cubilin gene variants warrants the question if urinary cubilin originates from podocytes [25]. Another finding that supports this hypothesis is that urinary VDBP level, a ligand of cubilin in PTEC, was not increased in this group despite an increase in cubilin shedding. We postulate that cubilin shedding in DM is due to early molecular alterations in podocytes.

In summary, we suggest that urinary cubilin can be a potential biomarker for predicting progressive DN in T1D. Our study population is comprised of patients followed for over 20 years as part of a large cohort. The major limitation of our study is its limited generalizability due to the small sample size. Although higher cubilin levels were associated with an increased likelihood of progression (odds ratio > 1), the small sample size limited the number of covariates that could be included in the model.

The possibility of protein degradation of urine samples during long‐term storage was minimized or prevented by avoiding multiple freeze‐thaw cycles before analysis. In addition, urine samples stored in the EDC have been successfully utilized in proteomics analysis previously [26].

Future studies are needed to validate urinary cubilin shedding as a biomarker in larger patient populations. Understanding the mechanism of cell signaling events that tie altered insulin signaling to membrane trafficking of cubilin in PTEC and podocytes is crucial in drug discovery in T1D. Furthermore, elucidating genotypic factors will assist in identifying patients that are at high risk for progressive DN.

## Funding

No funding was received for this manuscript.

## Disclosure

The authors have nothing to report.

## Conflicts of Interest

The authors declare no conflicts of interest.

## Supporting information


**Supporting Information** Additional supporting information can be found online in the Supporting Information section. The supporting table presents the levels of urinary biomarkers measured by ELISA, both unadjusted and normalized for creatinine.

## Data Availability

The data that support the findings of this study are available in the supporting information of this article.
