# Supplementary material for: A Pilot Study of Urinary Cubilin Shedding in Type 1 Diabetes Mellitus: A Potential Biomarker to Predict Nephropathy
Source: J Diabetes Res. 2026 Aug 21;2026:2291735. doi: 10.1155/jdr/2291735 (PMC13498853; doi:10.1155/jdr/2291735)
Supplement: Supplementary file 1 [file JDR-2026-2291735-s001.docx]

1)	SUPPLEMENTAL TABLE

|  | | Code | IL-8 | | NGAL | | VDBP | | Cubilin | |
| --- | --- | --- | --- | --- | --- | --- | --- | --- | --- | --- |
| Normal AER+GFR | | 25-61 | 55.83 | | 0 | | 1.5 | | 1.69 | |
| Normal AER+GFR | | 20-357 | 10.95 | | 27.3 | | 8.28 | | 2.48 | |
| Normal AER+GFR | | 19-79 | 69.97 | | 87.4 | | 0.69 | | 2.56 | |
| Normal AER+GFR | | 3-545 | 3.71 | | 118 | | 130.26 | | 2 | |
| Normal AER+GFR | | 5-508 | 8.51 | | 31.3 | | 21.4 | | 2.6 | |
| Normal AER+GFR | | 4-145 | 71.56 | | 88 | | 14.38 | | 3.15 | |
| Normal AER+GFR | | 15-23 | 54.23 | | 0 | | 0.49 | | 3.8 | |
| Normal AER+GFR | | 16-428 | 12.99 | | 26.8 | |  | | 3.02 | |
| Normal AER+GFR | | 17-677 | 3.73 | | 16.2 | | 18.37 | | 2.63 | |
| Proteinuria+Normal GFR | | 2-568 | 26.26 | | 0 | | 0.51 | | 3.31 | |
| Proteinuria+Normal GFR | | 6-816 | 4.34 | | 273 | | 0.61 | | 3.15 | |
| Proteinuria+Normal GFR | | 8-890 | 158.25 | | 12.9 | | 13.47 | | 3 | |
| Proteinuria+Normal GFR | | 13-1293 | 10.32 | | 82.7 | | 0.55 | | 3.08 | |
| Proteinuria+Normal GFR | | 14-1318 | 1.99 | | 9.66 | | 19.56 | | 2.6 | |
| Proteinuria+Normal GFR | | 23-224 | 2.34 | | 0 | | 0.63 | | 2.8 | |
| Proteinuria+Normal GFR | | 24-74 | 56.23 | | 0 | | 0.58 | | 2.11 | |
| Proteinuria+Normal GFR | | 27-1095 | 5.44 | | 17.4 | | 0.44 | | 2.71 | |
| Proteinuria+Normal GFR | | 18-155 | 2.56 | | 0 | | 4.51 | | 1.5 | |
| Decreased GFR | | 1-924 | 16.28 | | 12.1 | | 1.5 | | 2.89 | |
| Decreased GFR | | 7-781 | 5.09 | | 86.5 | | 0.8 | | 3.42 | |
| Decreased GFR | | 9-903 | 7.47 | | 45.7 | | 15.6 | | 2.65 | |
| Decreased GFR | | 10-993 | 161.14 | | 12.9 | | 20.69 | | 2.65 | |
| Decreased GFR | | 11-1221 | 4.09 | | 196 | | 3.09 | | 2.56 | |
| Decreased GFR | | 12-1005 | 3.73 | | 0 | | 0.69 | | 4.28 | |
| Decreased GFR | | 21-580 | 2.4 | | 0 | | 0.51 | | 2.7 | |
| Decreased GFR | | 22-356 | 7.57 | | 0 | | 3.38 | | 3.3 | |
| Decreased GFR | | 28-204 | 19.65 | | 30.4 | | 41.23 | | 2.49 | |
| Decreased GFR | | 26-720 | 11.44 | | 56.3 | | 16.87 | | 2.55 | |
| MCP | creatinine | creatinine(g | | IL-8/cre | | NGAL/cre | | VDBP/cre | | MCP/cre |
| 79.18 | 102 | 0.102 | | 547.35294 | | 0 | | 14.705882 | | 776.27451 |
| 77.53 | 102 | 0.102 | | 107.35294 | | 267.64706 | | 81.176471 | | 760.09804 |
| 166.71 | 330 | 0.33 | | 212.0303 | | 264.84848 | | 2.0909091 | | 505.18182 |
| 98.59 | 225.4 | 0.225 | | 16.488889 | | 524.44444 | | 578.93333 | | 438.17778 |
| 169.88 | 44.4 | 0.044 | | 193.40909 | | 711.36364 | | 486.36364 | | 3860.9091 |
| 161.2 | 118 | 0.118 | | 606.44068 | | 745.76271 | | 121.86441 | | 1366.1017 |
| 306.9 | 217 | 0.217 | | 249.90783 | | 0 | | 2.2580645 | | 1414.2857 |
| 264.18 | 141.4 | 0.141 | | 92.12766 | | 190.07092 | | 0 | | 1873.617 |
| 338.13 | 95 | 0.095 | | 39.263158 | | 170.52632 | | 193.36842 | | 3559.2632 |
| 22.81 | 96 | 0.096 | | 273.54167 | | 0 | | 5.3125 | | 237.60417 |
| 118.01 | 220 | 0.22 | | 19.727273 | | 1240.9091 | | 2.7727273 | | 536.40909 |
| 197.84 | 102 | 0.102 | | 1551.4706 | | 126.47059 | | 132.05882 | | 1939.6078 |
| 80.07 | 36.4 | 0.036 | | 286.66667 | | 2297.2222 | | 15.277778 | | 2224.1667 |
| 54.7 | 28.2 | 0.028 | | 71.071429 | | 345 | | 698.57143 | | 1953.5714 |
| 6.15 | 66 | 0.066 | | 35.454545 | | 0 | | 9.5454545 | | 93.181818 |
| 18.56 | 225.4 | 0.225 | | 249.91111 | | 0 | | 2.5777778 | | 82.488889 |
| 120.86 | 217.4 | 0.217 | | 25.069124 | | 80.184332 | | 2.0276498 | | 556.95853 |
| 21.07 | 64 | 0.064 | | 40 | | 0 | | 70.46875 | | 329.21875 |
| 39.16 | 28 | 0.028 | | 581.42857 | | 432.14286 | | 53.571429 | | 1398.5714 |
| 161.79 | 155 | 0.155 | | 32.83871 | | 558.06452 | | 5.1612903 | | 1043.8065 |
| 76.78 | 78 | 0.078 | | 95.769231 | | 585.89744 | | 200 | | 984.35897 |
| 29.41 | 42 | 0.042 | | 3836.6667 | | 307.14286 | | 492.61905 | | 700.2381 |
| 95.07 | 86.8 | 0.0868 | | 47.119816 | | 2258.0645 | | 35.599078 | | 1095.2765 |
| 20.99 | 77.4 | 0.077 | | 48.441558 | | 0 | | 8.961039 | | 272.5974 |
| 20.35 | 63.4 | 0.063 | | 38.095238 | | 0 | | 8.0952381 | | 323.01587 |
| 163.78 | 58 | 0.058 | | 130.51724 | | 0 | | 58.275862 | | 2823.7931 |
| 74.31 | 148 | 0.148 | | 132.77027 | | 205.40541 | | 278.58108 | | 502.09459 |
| 11.48 | 78.6 | 0.078 | | 721.794 | | 721.794 | | 216.282 | | 147.179 |
| cubilin/cre | | | | | | | | | | |
| 16.568627 | | | | | | | | | | |
| 24.313725 | | | | | | | | | | |
| 7.7575758 | | | | | | | | | | |
| 8.8888889 | | | | | | | | | | |
| 59.090909 | | | | | | | | | | |
| 26.694915 | | | | | | | | | | |
| 17.511521 | | | | | | | | | | |
| 21.41844 | | | | | | | | | | |
| 27.684211 | | | | | | | | | | |
| 34.479167 | | | | | | | | | | |
| 14.318182 | | | | | | | | | | |
| 29.411765 | | | | | | | | | | |
| 85.555556 | | | | | | | | | | |
| 92.857143 | | | | | | | | | | |
| 42.424242 | | | | | | | | | | |
| 9.3777778 | | | | | | | | | | |
| 12.488479 | | | | | | | | | | |
| 23.4375 | | | | | | | | | | |
| 103.21429 | | | | | | | | | | |
| 22.064516 | | | | | | | | | | |
| 33.974359 | | | | | | | | | | |
| 63.095238 | | | | | | | | | | |
| 29.493088 | | | | | | | | | | |
| 55.584416 | | | | | | | | | | |
| 42.857143 | | | | | | | | | | |
| 56.896552 | | | | | | | | | | |
| 16.824324 | | | | | | | | | | |
| 32.692 | | | | | | | | | | |
